# An evaluative study of the benefits of participating in intergenerational playgroups in aged care for older people

**DOI:** 10.1186/1471-2318-14-109

**Published:** 2014-10-08

**Authors:** C Margaret Skropeta, Alf Colvin, Shannon Sladen

**Affiliations:** School of Science and Health University of Western Sydney, Locked Bag 1797, Penrith, NSW Australia; School of Science and Health University of Western Sydney, Locked Bag 1797, Penrith, NSW Australia; UnitingCare Ageing, 1Wetherill Street, PO BOX 89, Leichhardt, NSW Australia

**Keywords:** Intergenerational playgroups, Friendship, Meaningful activity, Connectedness, Dignity, Dementia

## Abstract

**Background:**

Intergenerational playgroups in aged care are limited and little is known about the perceptions of individuals who have participated in such programs. Most research is focused on intergenerational programs that involved two generations of people – young people and older people or young people and people with dementia reported the significant outcomes for each group of participants. In this study a number of generations participated in the intergenerational playgroup intervention that included older people, child carers who were parents, grandparents or nannies and children aged 0–4 years old. The objective of this study was to explore the benefits of participating in an intergenerational playgroup program IPP in an aged care facility.

**Methods:**

This mixed methods quantitative and qualitative design explored the benefits of participating in an intergenerational playgroup program IPP in aged care settings. The intervention is an intergenerational playgroup program (IPP) offered in the aged care facility where intergenerational socialisation and interaction occurred between different generations. The SF36 and Geriatric Depression Scale (GDS) were used to collect pre-test post test data. The qualitative interpretive research approach used semi-structured interviews to develop the descriptive interpretation of the intergenerational playgroup experience. Interviews were conducted with aged care residents and child carers.

**Results:**

The pre-test post-test results for the SF36 revealed a declining trend in one scale only energy/fatigue and no significant differences on the Geriatric Depression Scale GDS. The interview analyses revealed the following themes (1) intergenerational experiences, (2) two-way contributions, (3) friendships work, (4) personal growth, and (5) environmental considerations and nineteen subthemes were extracted to provide meanings.

**Conclusions:**

The IPP provided a successful innovative intergenerational program intervention where older people and people with dementia interacted and connected with a number of people from different generations. The IPP provided meaningful engagement for all participants considered important for self-esteem and the ability to participate fully in society. This allowed people to develop a sense of connectedness and friendships in a safe and secure environment. This increased the dignity of older people and people with dementia within the community and increased public awareness about the existing care and support services available to them.

## Background

Intergenerational playgroup program IPP provided the opportunity for meaningful engagement between the generations research into intergenerational programs demonstrates how participants made meaningful contributions to each other’s lives [1]. This innovative program involved older adults who were cognitively intact and impaired. There are a limited number of programs that have considered the possibility of inviting adults with dementia to interact with young children [2]. The IPP in aged care is a family and non-familial intergenerational program where aged care residents and playgroup participants are not necessarily related but in some cases maybe related. The inclusion of a non-familial intergenerational program is a relatively new treatment milieu in elder care [3] and recognizes that contact between young and older generations remains an integral part of most families. Family forms show greater diversity then in earlier times, reflecting the complex social and economic changes in society that result in a lack of contact between young and old [4].

Dementia is a common health condition in older people. In Australia demographic ageing will lead to an increase in the number and percentage of people who have dementia – in 2009 1.1% of the population had dementia and it is estimated that by 2050, 2.8% of the population will have dementia [5]. Presently more than 50% of residents in Australian Government –subsidised aged care facilities have dementia (85,227 out of 164,116 permanent residents with an ACFI assessment at 30 June, 2011) [6].

Playgroups NSW provide an informal session where mums, dads, grandparents, caregivers, children and babies can meet in a relaxed and friendly environment while providing child care. Usually the adults stay at the session to interact with other adults and to play and support their children [7]. In this study older people and people with dementia in aged care facilities and child carers and children who attend playgroup are bought together in the IPP. Therefore the exchange of the experience regarding the potentialities of intergenerational encounters should be encouraged and reinforced [8].

Characteristics of successful intergenerational programs demonstrate mutual benefits for participants; establish new perspectives for young and old participants; involve multiple generations and must include at least two non-adjacent such as two generations removed and non-familial generations indicating not immediate family; promote increased awareness and understanding between the younger and older generations and the growth of self-esteem for both generations; address social issues and policies relevant to those generations involved; include the elements of good programming planning and develop intergenerational relationships [8]. Intergenerational programs provide the opportunity for meaningful engagement between the generations, for activities to be meaningful for people with dementia they need to experience pleasure and enjoyment, a sense of connection and retain a sense of autonomy [9]. Activities can also create immediate pleasure, re-establish dignity, provide meaningful tasks, restore roles and enable friendships, be therapeutic, enhance quality of life, arrest mental decline and generate and maintain self-esteem [10, 11].

Intergenerational programs are important for both individual self-esteem and the ability to participate fully in society [12]. Social connectedness reflects the self in relation to others, it is the internal sense of belonging and is defined “as the subjective awareness of being in close relationships with the world” [13]. Based on the connections people make they can develop friendships and people diagnosed with dementia can clearly retain the ability to enjoy moments of genuine mutual support and consideration and can understand what is entailed in warm, mutually satisfying relationships including friendships [14, 15]. Declines in social functioning maybe a more direct outcome of the ways in which the person diagnosed with dementia is positioned and treated by healthy others including family as well as formal care givers such as staff members at day centres and residential homes [15]. The IPPs in this study are best described as social networks loosely bound by the interlinking among individuals as sets of people that can extend beyond the immediate social environment [15] of the aged care facility.

The concept of introducing Playgroup NSW groups into aged care communities is new and challenging as it involves the intergenerational socialisation and interaction of three or four distinct age groups of people which include: babies and children under four years old; the child’s carer who were a parent, grandparent or nanny and the older person who is a resident in the aged care facility. The intergenerational intervention was where the talents of ordinary people of different ages and vulnerabilities become available in different ways to interact with one another. The social relationships matter to the well-being of people of all ages in a variety of ways and are the basic building block of healthy development for both mental and physical health throughout the life course are affected by ties to other people [15]. The social engagement and relationships that aim to build ties between participants in this project reinforce what have been convincingly argued to be the important links that contribute to the well-being of self and society [16].

Playgroup NSW is a community organization, and lead agency for playgroups in NSW offering children and their carers’ opportunities to learn about their world, make friends and develop social skills while adults benefit from a time to talk, make friends, share experiences, learn about schools, pre-schools and community support networks [17]. Intergenerational programs have been successfully facilitated with older adults in settings ranging from child care centres to college classrooms but few programs considered the possibility of inviting adults with dementia to interact with young children [2]. Positive effects associated with intergenerational playgroups in aged care facilities have been demonstrated [18, 19]. The concept of bringing generations together to support the young and the old to foster interdependence and meet societal needs has become an increasingly popular idea which can be viewed as a dynamic process of program development that moves towards deeper, more sustainable interpersonal relationships and inter-organizational partnerships [1]. A number of intergenerational playgroups were developed in recent years in Western Australia to date have not been formally evaluated [18]. In Victoria an intergenerational playgroup was established and evaluated in a residential aged care facility [19]. The impacts of the intergenerational playgroup on aged care participants were: enjoyment, intergenerational interactions, reflection and reminiscence of childhood and parenting and changes in attitudes/expectations and perceptions of different generations and aged care facilities [19]. These findings indicate the need for intergenerational play groups within society as they have the potential to contribute to building of social capital and increased social interaction between generations.

The purpose of this study was to explore the benefits of participating in an IPP offered in aged care facilities. The participants in this study are aged care residents that include older people and people with dementia and intergenerational play group participants identified as child carers that include parents, grandparents, nannies and children.

### Theoretical framework

The theoretical framework for this study is drawn from the perspective of symbolic interactionism [20, 21] and rests on three premises, first that human beings act towards things on the basis of the meaning that things have for them; second that the meaning of such things is derived from or arises out of the social interaction that one has with others and third that these meanings are handled in and modified through, an interpretative process used by the person in dealing with things he/she encounter. This perspective allowed the exploration of the IPPs that included socialisation and interaction patterns between older people, people with dementia, child carer such as parents, grandparents and nannies. This analysis identifies and explains the subject meaning the intergenerational playgroup had for individual participants as they took on a range of different roles [21] within the context of the IPPs.

## Methods

A mixed methods study of participants who attended the IPP was carried out using a quantitative and qualitative approach to offer a broader understanding [22] of intergenerational playgroups offered in aged care. A mixed method approach [23, 24] was used to provide a comprehensive analysis of the intergenerational playgroups offered in aged care facilities. Quantitative data was collected from aged care participants on a range of instruments that included: SF-36 (RAND 36 – Item Survey 1.0) which has become the most extensively validated and used generic instrument for measuring quality of life [25, 26]; Geriatric Depression Scale (GDS) a depression assessment tool specifically designed for older people [27, 28]. Qualitative interpretive research approach used semi-structured interviews [24] during the intervention period with aged care residents and child carers participating in the IPP. The interviews used the same interview protocol to collect a broad range of relevant information about the IPP. Four open-ended questions focussed on the nature, experience and perceptions of the participants and one question related to their evaluation of the IPP on themselves. The interviews were 15 – 30 minutes in length and were audio tape and transcribed verbatim for analysis.

### IPP sites

Site 1 Low Care (Hostel), High Care (Nursing Home) in the Sydney Region, with 130 places with 55 beds available specifically for residents in need of dementia care; Site 2 Low Care (Hostel) Hunter Central Coast and New England Region with 45 places, this with 15 beds dedicated to support people with dementia; and Site 3 High Care (Nursing Home) in the Sydney Region with 63 places is a Chinese specific nursing home.

### Participants

A convenience sample [23] of older adults who are cognitively intact and impaired, child carers and children who attended the intergenerational playgroup programs offered in three UnitingCare Ageing facilities, included forty eight aged care residents 43 women and 5 men ranging in age from 68 to 101 years with a mean age of 85 years. At the post-test data collection five aged residents had withdrawn due to illness or moving to another facility and one was deceased. As the IPP was a timetabled program within each site, all participants exercised their personal choice to attend each session. Not every aged care person who attended the IPP participated in this study, their right not to participate was respected for example some aged care residents were fringe dweller observers, while others may have been waiting for a visitor to go to the café or waiting for a bus trip program.

A convenience sample [23] of 41 child carers made up of 28 parents, 9 grandparents and 4 paid nannies who bought 50 children 0–4 years old to the three UnitingCare Ageing sites offering the IPP.

### Screening of aged care residents

The Mini Mental Status Examination (MMSE) a valid and reliable instrument widely used to screen for cognitive impairment in older adults was used to identify aged care participants cognitive status [29, 30] in this project. Of the 48 aged care participants screened with the MMSE 7 (15%) had no cognitive impairment; 14 (28%) had mild dementia indicated the persons’ memory problems are becoming noticeable; 18 (38%) had moderate dementia indicated the person may experience confusion for most of the time and the person was unable to remember important things like the names of their grandchildren and 9 (19%) experienced severe dementia indicated the person was sketchy of past life events but largely unaware of recent events and experiences [31].

### The intervention – The intergenerational playgroup program IPP

The IPP is defined as a diversional therapy/leisure lifestyle program offered in an aged care facility. This program introduced intergenerational socialisation and interaction between three distinct generations: the residents in the aged care facility, the child carers (parents, grandparents and nannies) and the children 0–4 years old.

The aged care residents are the responsibility of the leisure life style staff of the aged care facility and the children are the responsibility of the child carer. The IPP was offered as a one and a half hour session per week in each aged care facility.

The lifestyle manager promoted the IPP to staff, residents, the local community and timetabled it. This was done via the electronic notice board throughout the aged care facility and the local community through media advertising in the local paper and general practitioners offices. The lifestyle manager ensured aged care facilities policies and procedures were followed, the Café was family friendly and all people on the aged care site had a Criminal Record Check and were registered in the Sign-in/Sign-out log for the program each day they attended.

The diversional therapists were responsible to facilitate interaction between the residents and the playgroup participants, ensure the therapeutic value of the activities in the program and serve morning tea to the residents. Child carers bought their own morning tea or purchased it from the onsite Café. The therapist introduced special events and community initiatives throughout the program such as an RSPCA Fundraiser which involved the residents and children making cup-cakes.

The IPP was developed in line with Playgroup NSW playgroup guidelines [17] and was supported by Playgroup NSW and advertised on their website. The leisure lifestyle coordinator consulted the coordinator of the playgroup NSW regarding the program design. The playgroup coordinator assumed responsibility for the playgroup activities offered in the program which included structured and unstructured play and learning experiences such as finger painting and was responsible for the purchase of toys and the care of the toys for playgroup.

### Data collection

Age care participants were initially identified by the primary carer/therapist as participants in the IPP as those residents who could give consent and those residents for whom, consent would have to be obtained from a family member or legal guardian. At site 3 because it was a Chinese specific aged care facility all information sheets and informed consent forms and quantitative data collection forms SF36, GDS and MMSE were translated into Chinese language. The researcher contacted the appropriate person and provided a verbal description of the study. This was followed up with a written description of the study sheet and the informed consent form. Once informed consent was obtained the quantitative pre-test SF-36 and GDS data was collected by the researchers and research assistants. The primary carer/therapist responsible for each of the nine residents with severe dementia assisted the resident reporting their pre-test and post-test SF-36 and GDS scores.

The researchers collected the qualitative interview data from the aged care participants and child carers during the IPP sessions which were interrupted by school holidays and public holidays. The post-test SF-36 and GDS data was collected on a 6 month plan.

The qualitative data collection ceased at site 1prior to a new playgroup coordinator taking over the program as the current playgroup coordinator returned to work and her son commenced pre-school. At site 2 all interviews were completed to coincide with the post-test quantitative data collection due the presentation of the IPP in the self-contained dementia ward. At site 3 the end of the interview data collection was influenced by Chinese culture within the site which impacted on the number of participants interviewed. There was a decrease in the number of child carers and aged care residents interviewed at this site.

Child carer participants were initially identified by the playgroup leader as participants in the IPP. The researchers invited the identified child carers to participate in this study and provided a verbal description of the study. This was followed by a written description of the study sheet and the informed consent form. A parent/guardian gave written consent for the children involved in this study. The researchers collected the qualitative interview data from the playgroup participants during the IPP sessions.

### Research questions

The questions for this study were developed based on two site visits to possible future IPP sites prior to the commencement of this project. Discussions were undertaken with older people, people with dementia, diversional therapy staff, playgroup coordinators and a group of potential playgroup participants. The five interview questions were developed to explored clinical practice and policy related to offering intergenerational playgroups in aged care settings. The five research questions included:

1. What do you think about your intergenerational playgroup experience?
2. Why do you come to the intergenerational playgroup?
3. Why do you continue to come to the intergenerational playgroup?
4. What type of friendships have you made at the intergenerational playgroup? And
5. What are some of the things which are happening for you?

The following probes were used to help the interviewee’s expand their experience and perceptions of the IPP:

More information: ‘Tell me about…” Re-clarification: “You mean…..” or “Could I clarify your last point?” End: “I think I have covered everything – Is there anything else you want to tell me?”

The interviews ranged from 15 minutes – 30 minutes in length. The interviews were recorded and transcribed for analysis. Collection of the interview data was standardized to the research questions protocol approved by University of Western Sydney ethics committee.

### Data analysis

A pre-test/post-test design was used to examine the SF36 and the Geriatric Depression Scale (GDS) results. A model of repeated measures general linear between groups was conducted to explore the pre-test/post-test SF36 and the Geriatric Depression Scale. Analyses were performed using SPSS, version 20 IBM Inc. Armonk, NY [32].

The interview data was collected through individual interviews with each participant. The interview data was recorded and transcribed verbatim. The recorded interviews were listened to by the two researchers. The interview transcriptions were reviewed twice by both researchers: the first time for understanding the context, and the second time to identify and record themes from within the data [23, 24]. Data derived from interviews was summarised under each question for each group of participants. For example all answers to Question 1 What do you think of your intergenerational playgroup experience? from aged care residents were put into one electronic file, from parent and nanny child carers were put into a second electronic file, from grandparent child carers were put into a third electronic file. This process continued for Questions 2–5. Each individual file was searched for themes and meanings were established. The 3 groups of themes and meaning for each question were bought together to be representative of all participants in this study in an attempt to increase the reliability of the data and its interpretations [24].

From the transcript thematic analysis, five themes were developed and nineteen subthemes were identified to provide meaning. Themes and categories were grouped and recorded in a systematic way to interpret the data.

### Ethical considerations

The Ethics Committee of the University Western Sydney, Australia (H9006) approved the study. All participants were required to give written consent to participate in this study.

Authors 1 and 2 are researchers in this project and do not occupy dual roles, as they are both senior lecturers at the University of Western Sydney and author 3 is the project does not occupy a dual role, as she is the leisure lifestyle coordinator at UnitingCare Ageing.

The ethics of this project were discussed in the three sites where the project was carried out because of the complexity of the participants involved in this study which included children under the age of sixteen years old, older people with varying degrees of cognitive impairment, parents, grandparents and nannies who may not have been the legal guardian and people who speak English as a second language. The complexities which arose increased the length of this study. Anonymity and confidentiality was discussed with each participant who was also provided with written information in accordance with the ethics approval. All names have been changed to ensure confidentiality.

Baseline data indicated the aged care residents experience a broad range of cognitive abilities.

## Results

The pre-test post-test results indicated that older person’s perceptions’ of their health was relatively stable over the six month duration of this study. Five themes with nineteen subthemes emerged from the qualitative data analysis.

### SF-36

The SF-36 pre-test and post-test item means see Table 1. Analysis of these results show a significant difference between the pre-test and post-test results indicated a decrease on *Scale 4 – Energy/fatigue F* (1, 31) = 10.957. p = .002.

**Table 1:** **SF-36 Pre-test and post-test item means (N = 43)**

SF-36 Sub-scales	Pre-Test	Post-Test
Physical functioning	77.2	73.4
Role limitations due to physical functioning	81.9	79.9
Bodily pain	75	77
General health	54	53.6
Energy/fatigue	59.8	47*
Social functioning	53.3	50
Role limitations due to emotional problems	80	78.1
Emotional well-being	62.8	57

Note: *Denotes a significant difference p < .05.

### Geriatric Depression Scale GDS

A paired-samples *t-*test was conducted to examine the pre-test and post-test GDS results. These results indicated there was no significant difference between the pre-test and post-test results. Pre-test (*M* = 3.09*, SD* = 2.93) to post-test (*M*2.63, *SD =* 2.16), *t* (42) = 1.43, *p* > .05 (two tailed .187).

### Interview data

Five themes emerged: (a) Intergenerational experiences; (b) Two-way contributions; (c) Friendship work; (d) Personal growth; and (e) Environmental considerations and were broken down into nineteen subthemes to give added meaning to the them see Table 2: Intergenerational playgroup themes and meanings. The themes and meanings are presented below and are exemplified by direct quotes from the interview data. The quotes were selected because they are representative of the data collected in this study.

**Table 2:** **Intergenerational playgroup themes and meanings**

Themes	Meanings
Intergenerational experiences	• Connection between people
	• Intergenerational exchange
	• Representative of community
	• Enjoyment
	• Helping others
	Child development
Two-way contributions	• A lack of family support
	• Routine in lifestyle
Friendship work	• Between mothers, grandparents, grandparents and parents, residents and children, residents and residents
	• Recognition of others
	• The support network
Personal growth	• Residents expectation
	• Child carer’s growth
	• Opportunities for learning
Environmental considerations	• The aged care facility
	• Indoors and outdoors
	• Safety for all
	• The café
	• The shed

### Intergenerational experiences

The first theme intergenerational experiences allowed people to make connections with each other often in the face of a lack of family contact. This intergenerational exchange leading to enjoyment, helping others and child development in an environment which could be representative of the mix of generations associated with community life. Participants encountered a range of experiences with each other and were able to emphasis the value of the IPP through their personal encounters with others. The connection participants made with others encouraged attendance at the IPP. Gloria a resident confided about her attendance,

*I think it* [the intergenerational playgroup] *is rather good. For us older people, we get connected with the young children. At this time of day, we haven’t got much to do at our place. So I’m happy to come down. I think it is a very good idea, something to do.*

The intergenerational exchange was the factor that attracted many to the intergenerational play group. Robyn a mother said,

*I think the interaction with the oldies is lovely for Henry and Peter lovely moments between my son, and my nephew and older residents. I think it’s really a nice experience.*

The IPP is representative of community. Participants demonstrated the personal value and their beliefs about family, community and society in a busy twenty first century. Sharon a mother of a two year old boy and a seven month old daughter commented,

*I love it. I think it is correct, it is the way it should be. We shouldn’t have just all children’s group and mother’s group together. We should be more representative of the community. Just really coming here and to have like people that are really ready and waiting and willing to hold your baby and chat and who would be genuinely interested. And I can see they get something out of it.*

Enjoyment was expressed as a rewarding experience associated with being in the company of young children, helping others and observing and appreciating child development. Thelma an aged care resident said,

*I think it is great. Love being among those beautiful little children, I love coming because I love to see the children. I just love coming to see the children. Makes me feel good, I used to mind children, a long time, a long time ago.*

Helping others carried the meaning that specific help was extended to family members and aged care resident participants. Many grandparents who attend the IPP as child carers did so in order to provide help to their own children for example Raymond a grandfather of twin girls explained,

*Our daughter and her husband live in this suburb and we live in next suburb and we look after the children once a week, on Thursdays. The other grandparents, who also live not too far away, look after them one other day of the week.*

In essence participants in the IPP recognized child development in the children. Blanch the grandmother of Sam said,

*I found it extremely rewarding, he has learnt to mix with older people, he has been exposed to manners and been quite and been helpful and for his first experience of helping people, it’s been lovely.*

### Two-way contributions

For the second theme two-way contributions meanings that go beyond a lack of family support to a lifestyle routine. Participants respected and valued each other’s involvement in the IPP. Jane, the playgroup coordinator stated,

*The thing that really drove me to it was because we didn’t have any family here and that what I wanted my children to have some relationship and contact with people, older people that was the real key.*

Young families who moved to the city from other countries, interstate or rural areas for employment often experienced a lack of family support. Rebecca a mother explained,

*I’m a person where my where my family is in Queensland so they don’t get the opportunity to play with my mother their grandmother, so it’s nice just to get that experience. I think it is fantastic not only a child gets the opportunity to interact with the older generation but also for the older generation to interact with the children.*

The value of this type of experience was confirmed by an aged care resident Glenda who was a former primary school teacher, she stated,

*That’s to me a tremendous benefit personally having the interaction with the children which I didn’t fully have with my grandchildren. So I’m actually looking for as much of this as I can possibly get.*

Most of the parents, nannies and grandparents who attended the IPP playgroup valued and respected the aged care residents. Susan a mother commented,

*I think it is wonderful. Especially if they* [the residents] *don’t have any family to come and see them, I think it is nice for them to come and see the children playing and just be around other people.*

For many participants the IPP provided a routine in life. Pam a grandmother said,

*Well it’s a good way for us to get out of the house and somewhere where we go regularly and they get to know that that is where we are going.*

### Friendship work

The third theme friendship work goes beyond the family situation and network support to a casual recognition of others. A variety of friendships developed between the participants in the IPP which ranged from recognizing people with a smile to meeting people in different venues away from the intergenerational play groups. Friendships developed amongst mothers, between grandparents and parents, residents and children and residents and parents/grandparents.

Elizabeth a resident commented,

*Well because there is very limited time and there is such an age group, it can’t be intimate but a least it is recognition of faces. They recognise my face and I recognise theirs and we smile.*

The environment of the IPP encouraged residents and their family members too interact within the aged care facility where social interaction occurred randomly.

Family and social supports strengthened for example a resident’s son Steven took his three and half year old daughter to the IPP to see his mum he said,

*I guess we are pretty new but we have met a few people here today. Mum of course she lives here with some of the other people here so we know them and we are meeting a few other people as well*.

Social interaction which displayed affection happened between children and residents which, was not always planned. Jane the playgroup leader commented,

*I guess it is really nice to see the familiar residents that come on a regular basis and I think I’m developing a relationship with them. Probably 80% of the time through my daughter because of their* [the residents] *interest in her, they love nursing her.*

Mary a grandmother commented on the development of casual friendships. She said,

*There* [friendships] *fairly casual, I have had a couple of long conversations with another carer who’s here I am a grandparent and he is a parent but we have common interests that we can discuss.*

### Personal growth

The fourth theme personal growth moves beyond resident’s expectations and child carer’s growth to opportunities for learning for both groups. The personal growth experiences were varied and depended upon who the individual participant was. A number of mothers reported they were having their second babies and preparing their two year old child to commence pre-school another mother, indicated she was going back to paid employment. Nancy a resident commented,

*I did not know what to expect when I entered the aged care facility as a resident. I thought I would be with all the old boilers but the intergenerational playgroup changed that idea, I have experienced fun.*

Others were more reflective of their IPP experience for example Gail a nanny commented,

*I do not have to hover over the kids*

Social support between mothers was a common reason for participation in the intergenerational playgroup. Carol another mother commented,

*I came because of my mother’s group really to continue those relationships*.

The opportunity for personal growth allowed individuals to reflect on the personal circumstances of their life and develop new ways to view these experiences. Charles a grandfather commented,

*There is a tremendous benefit for me and for the people like me being in this context. I am 63. I saw my parents died in 2007. My mother died over several years with a lot of pain. My father died relatively quickly of a brain tumour. What becomes important for people of my age is to prepare them-selves for what happens when they are going to be 20 years older.*

Young mothers also faced some of their unknown fears associated with aged care facilities. Libby a mother of two children said,

*Coming to these facilities, I had no idea, I thought they* [the facility] *might be run down or the residents sitting in chairs but what this facility gives to the elderly with dementia is amazing.*

### Environmental considerations

The fifth theme environmental considerations through the identified meanings moves away from the physical space of the aged care facility; indoors and outdoors, the café and shed to where there is recognized conceptual safety for all within the environment of the IPP.

The weather often predicted where playgroup activities occurred. On wet days playgroup activities were inside and in the covered outdoor area. On sunny days playgroup activities occurred indoors and outdoors. At two facilities there was a range of outdoor play equipment such as slippery dips, castles and swings, at one facility there was an outdoor toy shed fill with age appropriate toys such as dinkies, wheel burrows, balls, strollers and pulling trailers full of blocks. Indoor toys include plastic tables and chairs, kitchen toys and building toys, books blocks and a dress up box. The third centre did not have any outdoor play equipment.

Leanne a mother said,

*This playgroup is really well resourced, beautiful grounds, great to have some outside facilities for the kids to play as well as indoors. The café yah, it is cheap coz it is so subsidised for the pensioners. So there are a lot of pluses about the actual physical environment of this playgroup.*

And Kathy another mother said,

*The environment is very nice and it is safe. And you can get food both you and the child like. I have to say though because Jack is a really outdoor boy. We end up spending a lot of time outside and the residents tend to be inside when they are here. So he might go up and say Hi or do a high five or whatever but that’s it is not that much engagement.*

## Discussion

The baseline data MMSE scores indicated that 41 older people 85% who participated in the IPP experienced mild to severe forms of dementia [30, 31]. Despite the significant cognitive impairment of many aged care clients, practitioners have found their experiences interacting with and caring for children so ingrained that they remain able to interact appropriately and positively with children until late in the progress of a dementia illness [3]. Only one significant change in self-reported health status was obtained for participants as a result of participation in IPPs. The SF-36 revealed a significant decline in the SF-36 subscale energy/fatigue. This change in energy/fatigue may have occurred due to the general age of the participants where the mean age was 85 years old. This may suggest deterioration but the sample size is too small to confirm this. The SF-36 results in this study for a population with a mean age of 85 years are similar to a previous study where the SF-36 was used with a community based population over 65 years except in the area of energy/fatigue [26]. The GDS [28] results indicated there was no significant difference between the pre-test and post-test results indicating the relative stability of the of the aged care participants perceptions of their own health over time.

As an intergenerational program the IPP achieved a number of the essential characteristics identified [8] in successful intergenerational programs. These included: demonstrated mutual benefits such as making connections between people; new perspectives for children and aged care participants on the intergenerational exchange; involvement of multiple generations from more than two different age groups and generations not of one’s immediate family; promotion of increased awareness and understanding between the younger and older generations as they observed and reached out to one another.

The IPP provided an opportunity for meaningful programming [33] for all participants specifically the persons with dementia which often challenges family and professional caregivers [3]. The connections participants made in the intergenerational playgroup were valued and provided the residents with something to do in the aged care facility and experiences that were important for individual self-esteem and the ability to participate fully in society [9, 10] as participants believed the IPP experiences were representative of the community.

What drew many young mothers and their children to the IPP was their experience of a lack of family support and grandparents valued the routine of the IPP while meeting their original goal of attendance which was helping their own children manage work and family pressures consistent with other grandparents who are contributing to society more than ever as they invested time, money, love, attention and care in their grandchildren [34]. Continued attendance at the IPP lead to the development of friendships, while these friendships are judged as casual they did provide varying levels of support and connection [13].

Personal growth occurred across participants but most importantly the researchers believe the IPP increased the dignity of older people and people with dementia within the community. This enhanced dignity included greater public awareness of older people and about existing care and support services offered for them [9]. The three different centres provided a safe environment for the IPP participants and provided a range of suitable amenities.

### Methodological considerations

The theoretical frame work for this study was drawn from the perspective of symbolic interactionism has allowed us to develop a clearer understanding [20] of the interaction which takes place within a IPP and appreciate the meaning participants associated with their personal experiences in the IPP. The nature of the social interaction in the IPP was varied and it is important to recognise that socialisation is a lifelong process and we are socialised each time we enter a new group [21]. The IPP intervention provided a situation where participants could make appropriate responses.

Evidence of alternative explanations being sought is an issue in this study. The participant’s responses to the questions were largely positive this may be because the IPP was offered as one of number of alternative leisure lifestyle programs timetabled at the same time each week. The choice to attend this program for aged care residents appears to account for the lack of negative comment regarding the IPP.

The verification in qualitative research is important to establish credibility and trustworthiness [24] of the research process utilised. The validity of the themes and meanings in this study are strengthened as both researchers independently examined the interview data for themes and meanings for each question and then corroborated the evidence for the presented themes and meanings.

Only one semi-quantification comment was made by an aged care participant, who indicated that the *“IPP gave her something to do”* and may reflect how she judges other leisure lifestyle programs on offer in aged care facilities.

The trustworthiness of the qualitative data and interpretation was checked by peers informally reading this manuscript. This manuscript adheres to the qualitative research review guidelines RATS [35] to comply with BMC guidelines and details the relevance of the study questions, appropriateness of the qualitative method, transparency of procedures and the soundness of the interpretive approach.

## Conclusions

The perceived general health of older people and people with dementia remained stable over the period of this IPP intervention. However they experienced significant differences in energy and fatigue as the program progressed which may have been aged related. The IPP is appropriately classified as a successful intergenerational program [8] and as such provided meaningful activities to all participants. Aged care residents had something to do while child carer’s were delighted to watch the growth and development of the child/children they bought to the IPP. Older people, people with dementia, child carer’s and children connected with each other in the aged care environment which was viewed as safe and secure for all. Young parents and their children in the absence of their own family bought joy and satisfaction to older people and people with dementia while child carer’s valued the routine and rewards associated with the IPP.

The finding of this study may be important in aged care facilities as the IPP has the potential to provide an appropriate intergenerational program that benefits all participants while enhancing the dignity [12] of older people and people with dementia.

### Limitations of the study

Several limitations of this study are noted. First, this pilot study was established as a small evaluation study which is inherently limited because it did not have a control group and no demographic data was collected on the child carers and children. Second, the fact that the IPP’s were in operation for different time frames for example the site one was operational for at least four years, the **site** two was operational for eighteen months and site three had only been operating for six months, therefore the playgroup processes were not controlled in this study. Third the IPP evaluated were set up and developed on two different models the community model and the volunteer model. No attempt at generalization is being made in this study. Regardless of the lack of generalization, this study identified positive outcomes associated with IPP in aged care facilities.

Findings from this study provide a foundation for future research. Future research should include multiple intergenerational playgroups of equal numbers set up on the two different models identified in this study for comparison between the community based model and the volunteer model. The IPP’s involved in this evaluation are continuing and subsequent evaluation would be of benefit. Future research should include the identification of developmental benefits for child participants involved in IPP’s.

## Abbreviations

IPP: Intergenerational playgroup program
GDS: Geriatric Depression Scale
MMSE: Mini Mental Status Examination.
